# Inhibitor screening using microarray identifies the high capacity of neutralizing antibodies to Spike variants in SARS-CoV-2 infection and vaccination

**DOI:** 10.7150/thno.67038

**Published:** 2022-02-28

**Authors:** Xiaomei Zhang, Mei Zheng, Hongye Wang, Haijian Zhou, Te Liang, Jiahui Zhang, Jing Ren, Huoying Peng, Siping Li, Haodong Bian, Chundi Wei, Shangqi Yin, Chaonan He, Ying Han, Minghui Li, Xuexin Hou, Jie Zhang, Liangzhi Xie, Jing Lv, Biao Kan, Yajie Wang, Xiaobo Yu

**Affiliations:** 1 State Key Laboratory of Proteomics, Beijing Proteome Research Center, National Center for Protein Sciences-Beijing (PHOENIX Center), Beijing Institute of Lifeomics, Beijing, 102206, China.; 2 Department of Clinical Laboratory, Beijing Ditan Hospital, Capital Medical University, Beijing, 100102, China.; 3 Department of Research Ward, Beijing Ditan Hospital, Capital Medical University, Beijing, 100102, China.; 4 State Key Laboratory of Infectious Disease Prevention and Control, National Institute for Communicable Disease Control and Prevention, Chinese Center for Disease Control and Prevention, Beijing, 102206, China; 5 Joint Laboratory for Pathogen Identification of ICDC and Ditan Hospital, Beijing Ditan Hospital, Capital Medical University, Beijing, 100102, China; 6 Beijing Key Laboratory of Monoclonal Antibody Research and Development, Sino Biological, Inc., Beijing, 100176, China; 7 Gobond Testing Technology (Beijing) Co., Ltd., Beijing, 102629, China.

**Keywords:** SARS-CoV-2, microarray, neutralizing antibody, mutation, Spike

## Abstract

**Rationale:** Mutations of SARS-CoV-2, which is responsible for coronavirus disease 2019 (COVID-19), could impede drug development and reduce the efficacy of COVID-19 vaccines. Here, we developed a multiplexed Spike-ACE2 Inhibitor Screening (mSAIS) assay that can measure the neutralizing effect of antibodies across numerous variants of the coronavirus's Spike (S) protein simultaneously.

**Methods:** The SARS-CoV-2 spike variant protein microarrays were prepared by printing 72 S variants onto a chemically-modified glass slides. The neutralization potential of purified anti-S antibodies and serum from convalescent COVID-19 patients and vaccinees to S variants were assessed with the mSAIS assay.

**Results:** We identified new S mutations that are sensitive and resistant to neutralization. Serum from both infected and vaccinated groups with a high titer of neutralizing antibodies (NAbs) displayed a broader capacity to neutralize S variants than serum with low titer NAbs. These data were validated using serum from a large vaccinated cohort (n = 104) with a tiled S peptide microarray. In addition, similar results were obtained using a SARS-CoV-2 pseudovirus neutralization assay specific for wild-type S and five prevalent S variants (D614G, B.1.1.7, B.1.351, P.1, B.1.617.2), thus demonstrating that high antibody diversity is associated with high NAb titers.

**Conclusions:** Our results demonstrate the utility of the mSAIS platform in screening NAbs. Moreover, we show that heterogeneous antibody populations provide a more protective effect against S variants, which may help direct COVID-19 vaccine and drug development.

## Introduction

Coronavirus disease 2019 (COVID-19) is caused by the severe respiratory coronavirus 2 (SARS-CoV-2). As of February 2022, SARS-CoV-2 had infected about 406 million people and caused 5.8 million deaths worldwide 1. A key step in infection is viral entry, which is facilitated by the interaction between the SARS-CoV-2 Spike (S) protein via its receptor binding domain (RBD) (319 - 541 aa) with the human Angiotensin-Converting Enzyme 2 (ACE2) receptor. Thus, this interaction is a major focus in drug and vaccine development efforts 2, 3. Unfortunately, SARS-CoV-2 is mutating, with new variants emerging nearly every week that could impede drug development and reduce the efficacy of COVID-19 vaccines 3, 4.

Mutations in the S protein are of particular concern since they could enable SARS-CoV-2 to evade defense mechanisms that are elicited by COVID-19 vaccines and therapeutic antibodies 5, 6. For example, the D614G variant, which was first identified in July 2020, has a faster infection rate and higher viral load in the upper respiratory tract than the wild-type “Wuhan-Hu-1” strain 7, 8. It has since become one of the most prevalent strains. The B.1.1.7 variant (D614G, N501Y) is more infectious and may lead to increased mortality compared to the parental strain 9, 10. The B.1.351 and P.1 variants contain three RBD mutations at E484K, N501Y, and K417N or K417T, respectively. These mutations have shown resistance to neutralizing antibodies (NAbs) produced by convalescent COVID-19 patients and vaccinees that inhibit the wild-type Spike-ACE2 interaction 11-16. The vaccinees in these studies received the most popular vaccines worldwide, including mRNA-based COVID-19 vaccines (Moderna and Pfizer BioNTech) and a replication-deficient chimpanzee adenoviral vector COVID-19 vaccine (AstraZeneca). Similar results were obtained when testing a B.1.1.7 variant with an additional E484K mutation 15, 17. These studies highlight the importance of an assay that can measure the humoral response to S variants in developing effective therapeutic antibodies and vaccines for COVID-19 2, 18.

To address this urgent need, we developed a protein microarray for the high throughput, multiplexed detection of NAbs to SARS-CoV-2 S variants. This multiplexed Spike-ACE2 Inhibitor Screening (mSAIS) assay is simple to use, able to detect NAbs to numerous S variants simultaneously, requires minimal sample volume (i.e., 20 µL serum), and can be performed with common laboratory equipment. It could also be used with other potential neutralizing molecules (e.g., small molecules). To demonstrate the potential of the mSAIS assay, we assessed the neutralization potential of purified anti-S antibodies and serum from convalescent COVID-19 patients and vaccinees across 72 S variants. The sensitivity and resistance of the various S protein mutations to the NAbs were determined, and new escape mutations that are not targeted by vaccine-induced antibodies were identified**.** The neutralization capacity of high and low titer NAb samples was also compared. Our results were validated using a peptide-based microarray and SARS-CoV-2 pseudovirus neutralization assay.

## Results

### Development of the mSAIS assay

**Figure 1:**
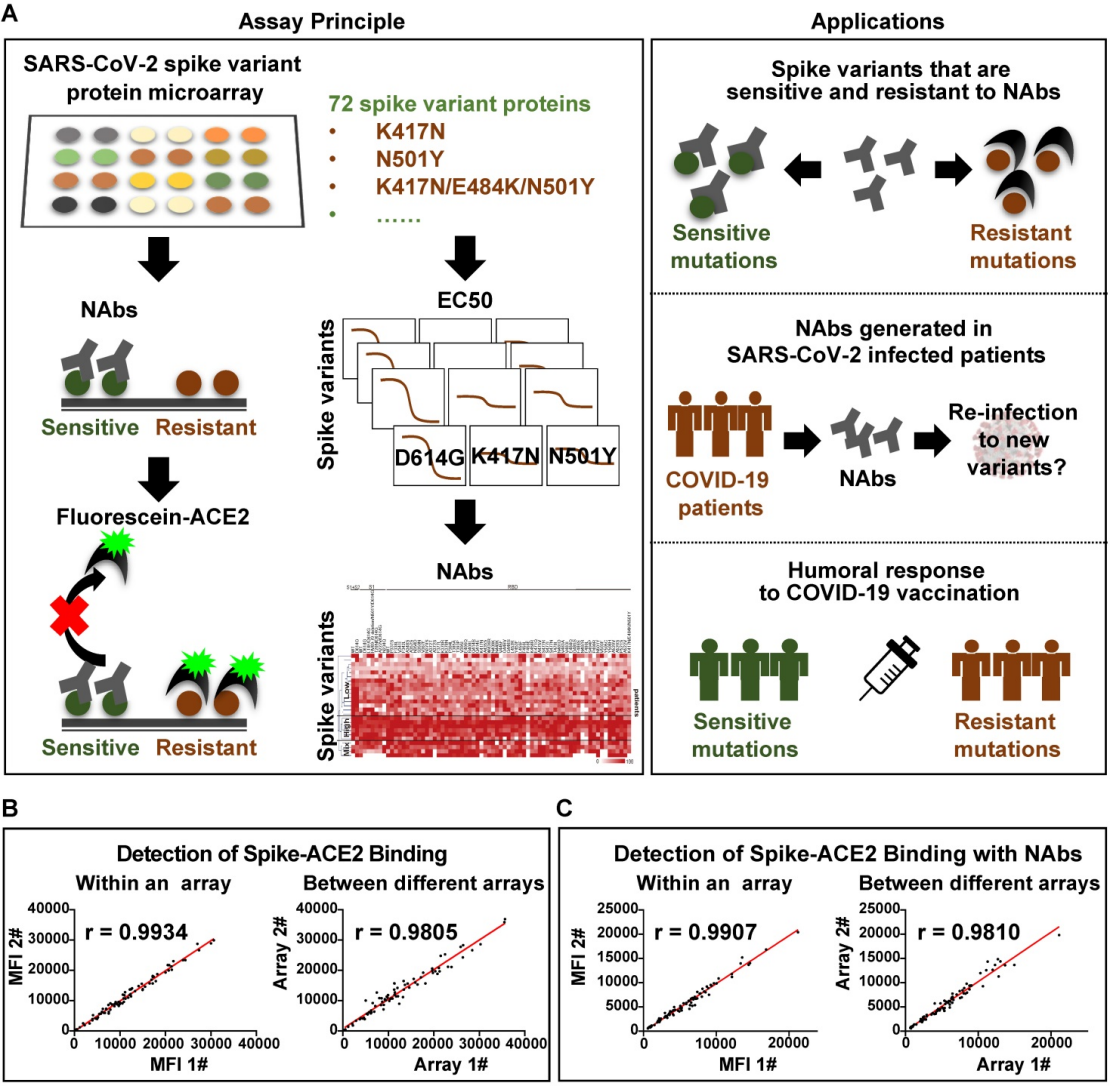
** Development of the multiplexed spike-ACE2 inhibitor screening (mSAIS) assay.** (A) A schematic illustration of the microarray-based mSAIS assay and potential biomedical applications; (B, C) Intra- and inter-array reproducibility of the mSAIS assay in the absence and presence of neutralizing antibodies, respectively. The *r* correlation of fluorescent signals within and between different arrays were calculated.

The mSAIS assay enables the simultaneous screening of various potential neutralizing molecules across numerous SARS-CoV-2 S variants within 2 h (Figure 1A). Here, 72 S protein variants with a polyhistidine tag at the C-terminus were expressed in the human embryonic kidney 293 (HEK293) cell line (Figure S1), purified, and printed onto a chemically-modified glass slide as previously described 19, 20. The printed S protein variants were selected containing mutations from the COVID-19 virus mutation tracker database 21 (Figure S2) and literature 22, 23, and included 2 variants with mutations in the S protein's subunit 1 (S1) and subunit 2 (S2) domains, 7 variants with mutations in S1, and 63 variants with mutations in the RBD 21. The worldwide cumulative prevalence of these mutations was tracked in the Global Initiative on Sharing Avian Influenza Data (GISAID) database as of December 28, 2021 (Table S1). The most prevalent D614G mutation and some mutations of interest (i.e., N501Y, L452R, K417N, N439K, S477N, S494P) were among the variants printed. In addition, two negative controls and one positive control (SARS-CoV-2 wild-type RBD) were printed on the array (Figure S3). The negative controls included on the array were the SARS-CoV-2 Nucleocapsid (N) protein and the RBD of the Middle East respiratory syndrome coronavirus 2 (MERS-CoV-2). Following printing, the sample (e.g., NAb) and a Cy5-labeled extracellular domain of ACE2 were sequentially incubated on the array with alternated wash steps to remove unbound sample and ACE2. Thus, S-ACE2 complex formation resulted in fluorescence whereas neutralizing samples decreased fluorescence.

Before using the mSAIS assay to screen samples, the reproducibility of the array and the binding of the S variants with ACE2 in the presence and absence of NAbs were evaluated. The results show that the intra- and inter-array *r* correlations for each step of the assay were 0.99 and 0.98, respectively (Figure S4, Figure 1B-C). Furthermore, the calculated neutralizing capacity of 13 serum samples from vaccinees obtained with the mSAIS assay and the live SARS-CoV-2 was compared. The *r* correlation between the two approaches was 0.82 (Figure S5), demonstrating that the mSAIS assay has high reproducibility and is a feasible approach for screening potential neutralizing molecules. It is also important to note that working with live SARS-CoV-2 requires biosafety level 3 (BSL3) facilities whereas the mSAIS assay can be performed safely at BSL1 or BSL2 depending on the nature of the tested samples.

### Characterizing ACE2 interactions with Spike variants using the mSAIS assay

**Figure 2:**
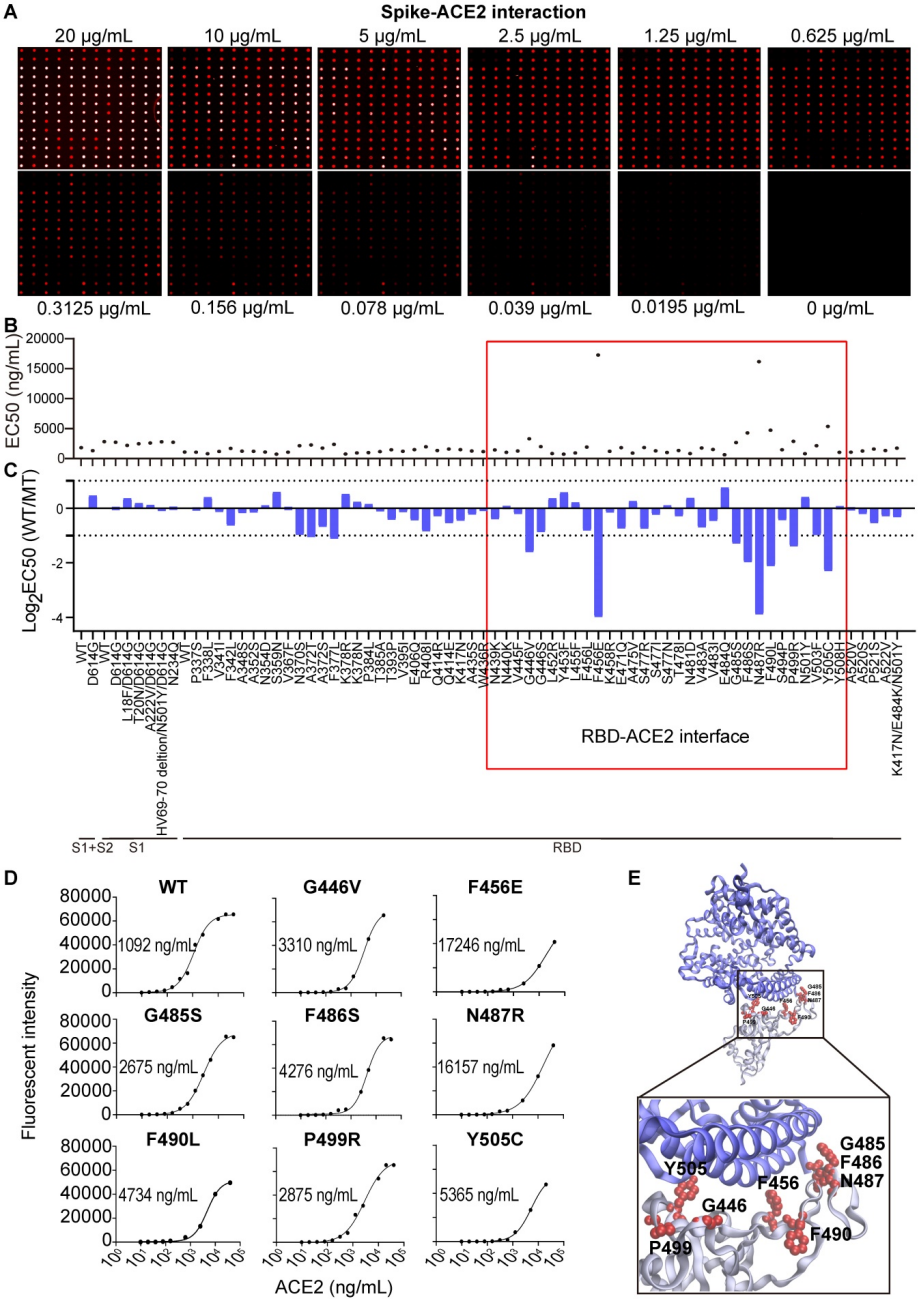
** SARS-CoV-2 Spike-ACE2 interaction mapping across different S variants using the Spike variant protein microarray.** (A) Detection of ACE2 binding at different concentrations to Spike variants immobilized on the array. Cy5-labeled ACE2 was added to the array at two-fold serial dilutions from 0 to 20 µg/mL, and the binding of ACE2 to the array was measured via the Cy5 tag. The EC50 was calculated using GraphPad Prism software 8.3 based on the fluorescent signal intensity. (B) Distribution of S-ACE2 binding affinities (EC50) across different SARS-CoV-2 S variants. (C) Fold changes of binding affinity between the wild-type and mutated S proteins. The x-axis represents the S variants and the y-axis represents the log2 transformed EC50 ratio between the wild-type and mutated S proteins. The red frame indicates the mutations that located at the RBD/ACE2 binding interface. (D) shows the binding curves of S variants different concentrations of Cy5 labeled ACE2. The EC50s are indicated. (E) Structural analysis of escape mutations within the RBD (gray color) that interact with ACE2 (blue color). RBD mutations (PDB ID: 6M0J) are labeled in red.

By testing different amounts of purified antibodies or serum, the half maximal inhibitory concentration (IC50) or effective concentration (EC50) can be determined, respectively, and the results visualized via fluorescence. To characterize the binding interactions between ACE2 and S variants, we incubated the microarray with fluorescent-labeled ACE2 at different concentrations. When the concentration of ACE2 was decreased (≤ 2.5 µg/mL), the fluorescent signal across the different S variants varied, indicating that the binding affinities of ACE2 to the S variants are not the same (Figure 2A). Indeed, the calculation of the EC50 for the S variants with mutations between residues 234 - 614 shows that the binding affinity (EC50) ranges from 0.65 to 17.25 µg/mL. ACE2 had the lowest binding affinity to two S variants, F465E and N487R, with an EC50 of 16.16 and 17.25 µg/mL, respectively (Figure 2B).

The EC50 ratios between the wild-type and variant S proteins were calculated and log2 transformed (Figure 2C). Using a 2-fold minimum as the selection criteria, 10 mutations that weaken the S-ACE2 interaction were identified, including A372T, F377L, G446V, F456E, G485S, F486S, N487R, F490L, P499R and Y505C (Figure 2C-D). Consistent with the observation that the trimerized form of the D614G S protein has an increased binding affinity to ACE2 21, we also found the D614G mutation modestly improved the binding of S-ACE2 in this study. An additional five mutations had ≤ 2-fold increased binding to human ACE2 (Figure 2C-D): L452R (in B.1.427/429 and B.1.617), Y453F (in B.1.1.298), E484Q (in B.1.617.1 and B.1.617.3) and N501Y (in B.1.1.529, B.1.1.7, B.1.351 and P.1). Thus, these mutations may facilitate the SARS-CoV-2 infection and escape from the NAbs 24-27.

Interestingly, 8/10 (80%) mutations that decreased the ability of the S protein to bind ACE2 (G446V, F456E, G485S, F486S, N487R, F490L, P499R, Y505C) are located at the RBD-ACE2 interaction interface (Figure 2E). Moreover, F456E and N487R had the weakest ACE2 binding affinities. These results further support the importance of the RBD domain in antibody neutralization 3, 28, 29.

### Neutralizing activity of anti-RBD antibodies to Spike variants

**Figure 3:**
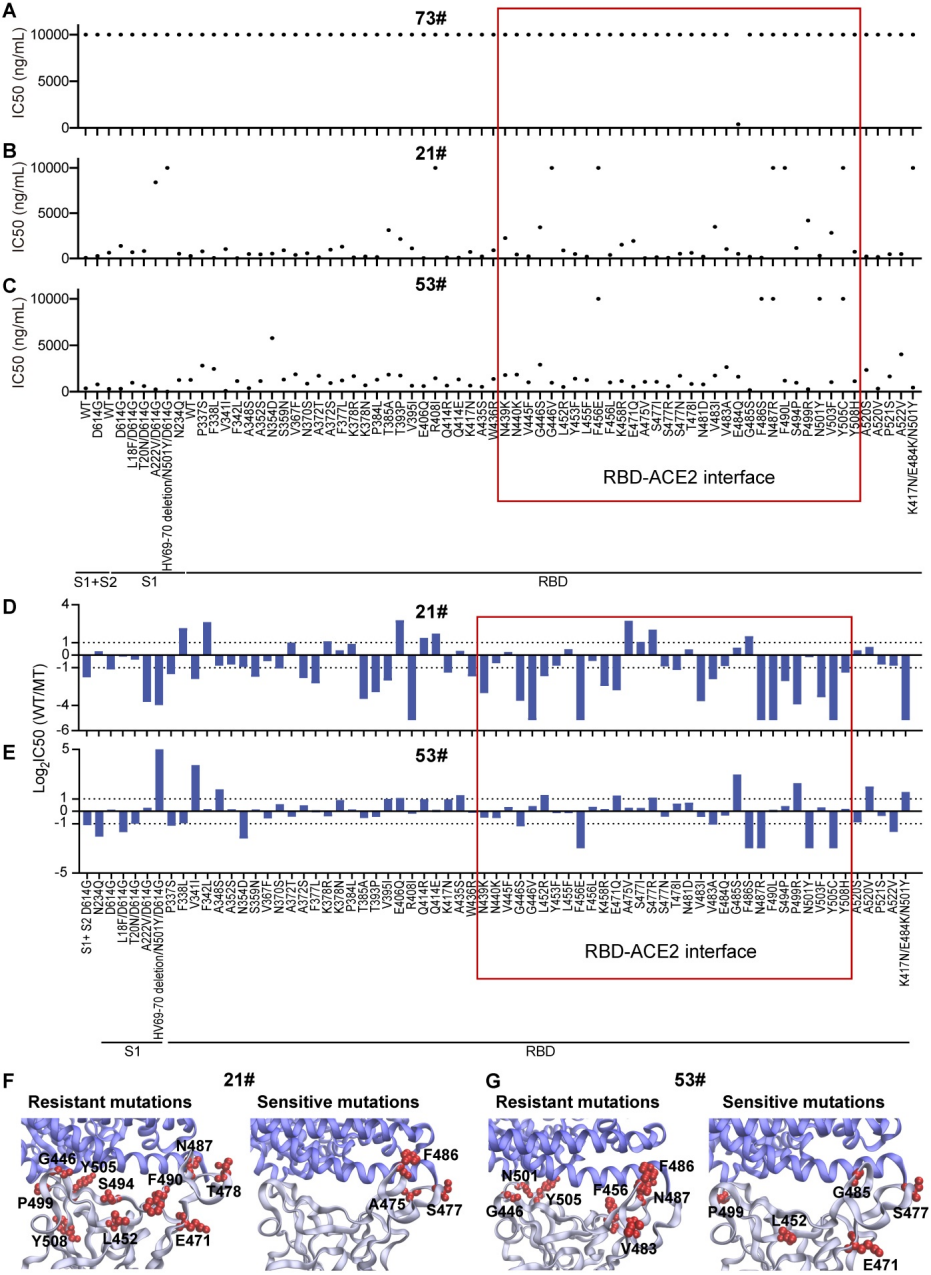
** Heterogeneous responses of purified anti-RBD antibodies to SARS-CoV-2 mutations.** (A-C) Distribution of neutralization activity (IC50) of anti-RBD antibodies #73, #21, and #53 to the S variants measured by the mSAIS assay, respectively. The x-axis represents the S variants and the y-axis represents the neutralization activity (IC50) of the antibody to each spike variant. (D, E) Fold changes of the neutralization activity (IC50) of antibodies #21 and #53 between the wild-type and mutant S proteins, respectively. The x-axis represents the S variants and the y-axis represents the log2 transformed IC50 ratio between the wild-type and mutated S proteins. (F, G) Structural analysis of mutations located at the RBD-ACE2 interaction interface that are resistant and sensitive to antibodies #21 and #53, respectively. The resistant and sensitive mutations are defined as ≥ 2-fold decrease or increase in neutralization activity (IC50) compared with the wild-type S protein. RBD is colored in gray, ACE2 is colored in blue, and RBD mutations are labeled in red (PDB ID: 6M0J). The red frame indicates the mutations at the RBD/ACE2 binding interface.

Three antibodies that bind to the S protein's RBD (Figure S6) were tested with the mSAIS assay, including a mouse monoclonal antibody #73, a rabbit polyclonal antibody #21, and a rabbit monoclonal antibody #53 (Figure 3). The IC50 of antibodies #21 and #53 to the S variants range from 42.04-10,000 ng/mL and 0.00063-10,000 ng/mL, respectively (Figure 3B-C). However, antibody #73 did not inhibit the S-ACE2 interaction (IC50 ≥ 10,000 ng/mL) (Figure 3A), indicating that not all anti-RBD antibodies have neutralizing activity. Next, we identified the resistant and sensitive mutations that decrease and increase the ability of antibodies to inhibit S-ACE2 interactions, respectively. A mutation that did inhibit S-ACE2 binding at all was categorized as a “complete resistance” mutation. Our data indicate that 8 S variants had complete resistance to antibody #21, including R408I, HV69-70 deletion/N501Y/D614G (B.1.1.7 strain), G446V, F456E, N487R, F490L, Y505C, and K417N/E484K/N501Y (B.1.351 strain) (Figure 3B). Two other studies also observed similar results for the B.1.351 strain 12, 30. Five mutations (F456E, F486S, N487R, N501Y, Y505C) (Figure 3C) had complete resistance to antibody #53.

Next, the IC50 ratios of antibodies #21 and #53 between the wild-type and S protein variants were calculated and log2 transformed. Using a two-fold difference in signal compared to the wild-type S protein as the criteria, 33 variants showed resistance to neutralization with antibody #21 (Figure 3D). Seventeen of the 33 (51.5%) mutations are located between residues 437 and 508, which is the region of the RBD that physically interacts with ACE2 31 (Figure 3D and 3F, Figure S7). Four of the ten (40%) sensitive mutations are also located within the RBD interface (Figure 3D and 3F).

Antibody #53 had reduced binding to 13 resistant mutations, in which seven (53.85%) mutations are located within the interface of the RBD-ACE2 interaction (Figure 3E and 3G). Interestingly, six (46.15%) sensitive mutations are not located within the RBD-ACE2 interface (Figure 3E and 3G), which indicates that binding to non-RBD binding epitopes can also have neutralization effects 22, 32.

### Heterogeneous reaction of serological NAbs to S variants in convalescent COVID-19 patients

**Figure 4:**
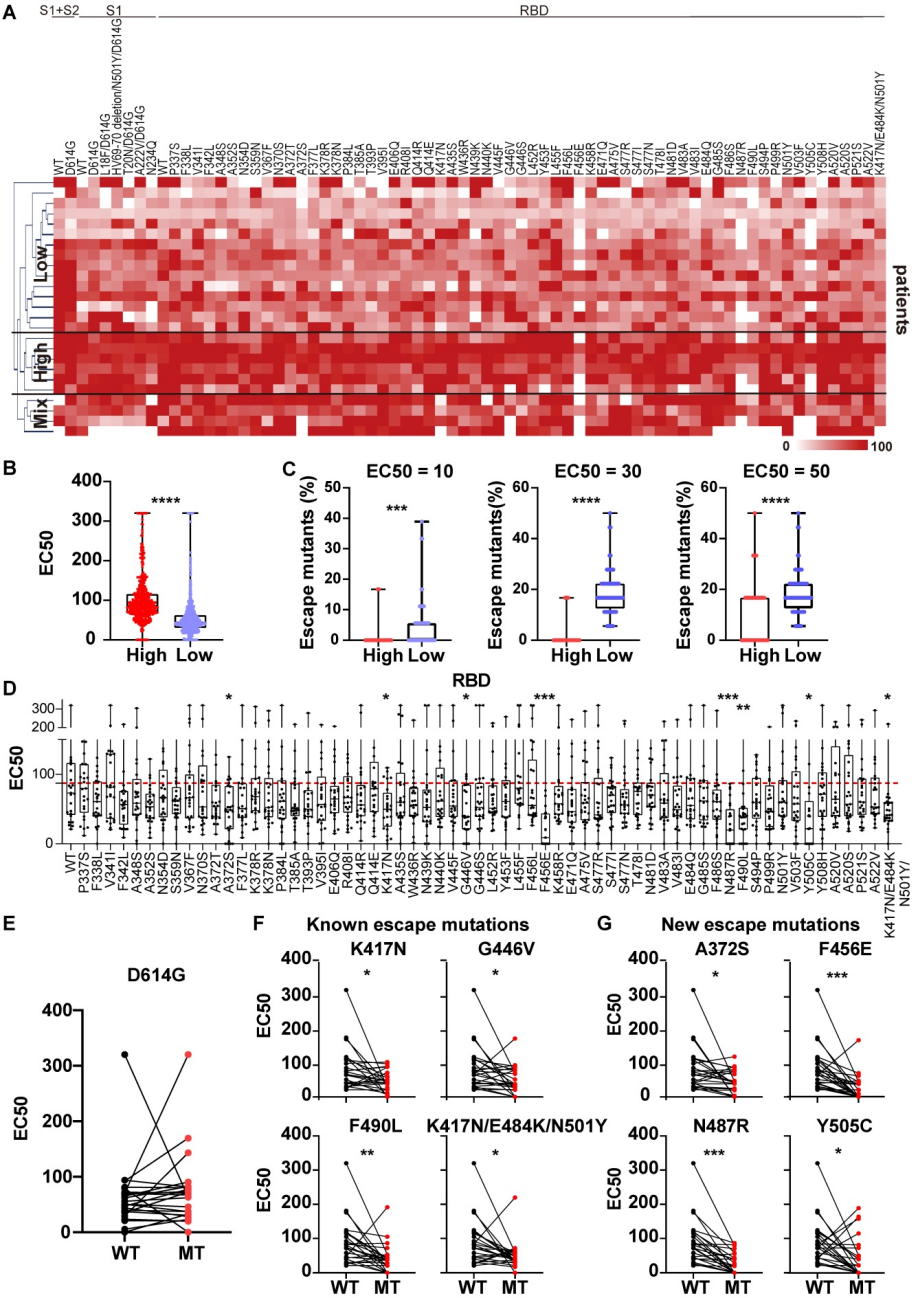
** Heterogeneous responses of serological NAbs to SARS-CoV-2 S mutations in convalescent COVID-19 patients.** (A) Hierarchical cluster analysis of serum NAbs to S variants from convalescent COVID-19 patients (n = 25) detected with the mSAIS assay. The rainbow color from white to red correspond to the EC50 from low to high, respectively. (B) Neutralization (EC50) of convalescent serum against S variants in the high NAb-titer group (n = 6) was compared to the low NAb-titer group (n = 15). (C) Comparison of escape mutations between convalescent COVID-19 patients with high and low NAb titers with EC50 thresholds set at 10, 30 and 50, respectively. (D) Neutralizing antibody titers (EC50) of convalescent sera against SARS-CoV-2 S-RBD variants using the mSAIS assay. The red dotted line indicates the median EC50 of serum to wild-type RBD. (E) Comparison of NAb titers between the wild-type and D614G S proteins. (F) Comparison of NAb titers between the wild-type S protein and S variants with known escape mutations. (G) Comparison of NAb titers between the wild-type S protein and S variants with newly identified escape mutations. Escape mutations have a significantly lower EC50 than wild-type mutations as determined using an unpaired t test with a p-value ≤ 0.05. *, P < 0.05; **, P < 0.01; ***, P < 0.001; ****, P < 0.0001.

We next screened the serum from 25 COVID-19 patients who had recovered from SARS-CoV-2 infection with the mSAIS assay (Table S2). The heat map displayed in Figure 4A showed the EC50 of convalescent serum against S variants. Using hierarchical cluster analysis, the convalescent patients clustered into three groups based on their NAb titers: high, low, and mixed (Figure 4A-B). After determining the percentage of escape mutations under three different EC50 thresholds (10, 30, 50), it was observed that the number of escape mutations were fewer in the high-titer NAb group than the low-titer NAb group (Figure 4C).

Next, the convalescent serum NAbs to wild-type and mutant S proteins were compared (Figure 4D, Figure S8). The convalescent serum showed similar neutralization activity against D614G variant and wild-type S proteins (Figure 4E, Figure S8). Using statistical analysis, the neutralization of convalescent serum NAbs to eight mutated proteins was significantly decreased, including 4 known escape mutations [K417N, G446V, F490L, K417N/E484K/N501Y (B.1.351 strain)] and 4 newly identified escape mutations (A372S, F456E, N487R, Y505C) (Figure 4F-G) 22, 30, 33, 34. Notably, structural analyses show that 5 escape mutations (K417N/E484K/N501Y, G446V, F456E, N487R, F490L) are located within the interface of RBD-ACE2 interaction, indicating that this RBD subdomain is important in neutralizing SARS-CoV-2 infection 19. All these results demonstrate the heterogeneous reactivity of NAbs produced by COVID-19 patients across S variants.

### Heterogeneous reactivity of NAbs produced by vaccinees to S variants

**Figure 5:**
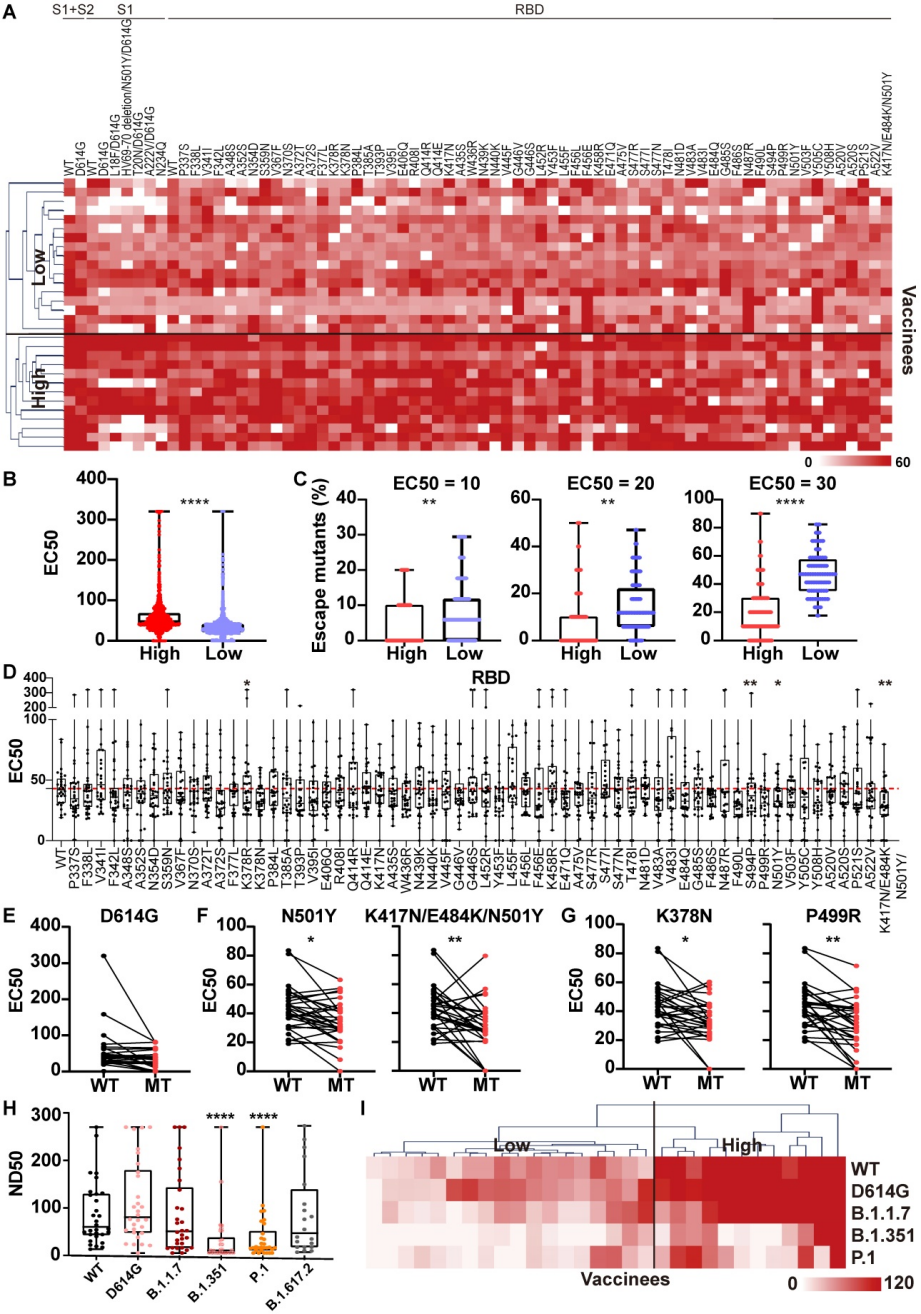
** Heterogeneous responses of serological NAbs to the SARS-CoV-2 S mutations in vaccinated individuals.** (A) Hierarchical cluster analysis of serum NAbs to S variants in vaccinees (n = 30) detected with the mSAIS assay. The rainbow color from white to red corresponds to the EC50 from low to high, respectively. (B) Neutralization (EC50) of vaccinee serum against S variants in the high NAb-titer group (n = 13) compared to the low NAb-titer group (n = 17). Data are median with all points. (C) Comparison of escape mutations between vaccinees with high and low NAb titers with EC50 thresholds set at 10, 20 and 30, respectively. (D) Neutralizing antibody titers (EC50) of vaccinee sera against SARS-CoV-2 S-RBD variants using the mSAIS assay. The red dotted line indicates the median EC50 of serum to wild type RBD. (E) Comparison of NAb titers between the wild-type and D614G S proteins. (F) Comparison of NAb titers between the wild-type S protein and S variants with known escape mutations. (G) Comparison of NAb titers between the wild-type S protein and S variants with newly-identified escape mutations. (H) Detection of NAb titers of vaccinee serum (n = 30, Table S3) to prevalent SARS-CoV-2 variants using the pseudovirus neutralization assay. (I) Hierarchical cluster analysis of the NAbs titers of vaccinee serum against prevalent SARS-CoV-2 variants. The rainbow color from white to red correspond the NAb titers from low to high, respectively. Escape mutations have a significantly lower EC50 than wild-type mutations as determined using an unpaired t test with a p-value ≤ 0.05. *, P < 0.05; **, P < 0.01; ***, P < 0.001; ****, P < 0.0001.

The mSAIS assay was next used to measure the neutralizing effect of serum from 30 vaccinees 1 month after the second dose of inactivated vaccine (Table S3). The NAb titers of vaccinee serum were visualized with a heat map (Figure 5A), which show that the vaccinee serum exhibited neutralization to most S variants. Using hierarchical cluster analysis, the vaccinees consistently clustered into two groups based on the level of their NAb titer: low and high (Figure 5A-B). Using three EC50 thresholds (10, 20, 30), high-titer NAb group had a lower number of escape mutations when compared to low-titer NAb group (Figure 5C).

Next, the NAb titers of vaccinee serum against all S variants were ascertained (Figure 5D, Figure S9). Like the convalescent COVID-19 patients, the D614G variant did not alter the neutralizing effect of the NAbs compared to wild-type S1+S2, S2, and RBD (Figure 5E, Figure S9). Using statistical analysis, four escape mutations were identified, including 2 known (N501Y, K417N/E484K/N501Y) and 2 new (K378N, P499R) mutations (Figure 5F-G) 34. To assess the longevity of NAb production following vaccination with an inactivated vaccine, we measured the serum NAbs to S variants six months after the second dose using the mSAIS assay of 25 vaccinees (Table S4). The titers of serum NAbs to both WT and S variants significantly decreased (Figure S10), suggesting the necessity of a booster dose.

To validate the results obtained with the mSAIS assay, we analyzed the neutralizing activity of vaccinee serum NAbs against wild-type, D614G, B.1.1.7 (alpha), B.1.351 (beta), P.1, B.1.617.2 (delta) S proteins using a SARS-CoV-2 pseudovirus neutralization assay. There was no significant difference of serum NAb titers between wild-type and the variants of B.1.617.2, B.1.1.7, and D614G. However, the NAbs titers to the B.1.351 and P.1 variants significantly decreased (Figure 5H), which is consistent with previous studies 35, 36. The results for the D614G, B.1.1.7, B.1.351 variants aligned with the data obtained with the mSAIS assay. The NAb titers of vaccinee serum (ND50) measured by pseudovirus neutralization assay and the vaccinees were also separated into high- and low-titer NAb groups with hierarchical clustering as displayed with a heat map (Figure 5I), further supporting the data obtained with the mSAIS assay (Figure 5C).

### High titer of NAbs contain anti-Spike antibodies with diverse binding epitopes

Two recent studies with rhesus macaques have shown that a high titer of NAbs produced in response to vaccination (DNA-based or adenovirus serotype 26 (Ad26) vector-based) provided good protection against infection when challenged with SARS-CoV-2 37, 38. In another study, NAb titers were significantly higher in vaccinees who had COVID-19 previously than vaccinees who never had COVID-19. Moreover, the vaccinees who had been infected with SARS-CoV-2 elicited NAbs that boosted neutralizing titers against S variants 39.

**Figure 6:**
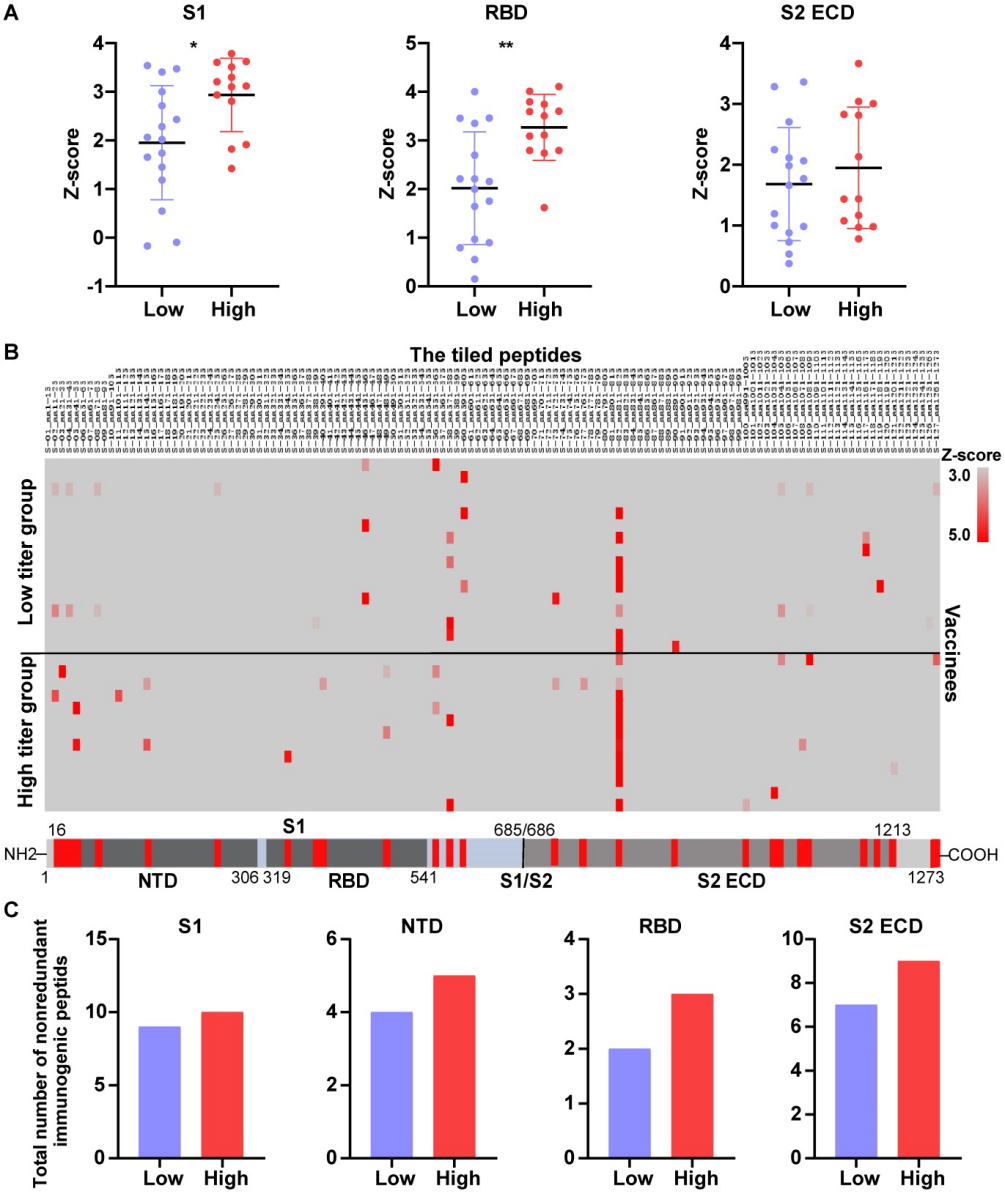
** The NAb titer is associated with the diversity of anti-S antibodies in the serum of vaccinees.** (A) A comparison of levels of antibodies that target the S1, RBD and S2 ECD between the high (n = 13) and low (n = 17) NAb titer groups. The data were measured by the SARS-CoV-2 proteome microarray and are plotted as the mean with the error bars representing the standard deviation (SD). (B) Identification of vaccine-induced IgG antibodies to epitopes on the S protein. (C) The total number of nonredundant immunogenic peptides on the S protein in the high (n = 13) and low (n = 17) NAb titer groups. A p-value (p) ≤ 0.05 calculated with the Mann-Whitney test was considered significant. *, P < 0.05; **, P < 0.01. NTD, N-terminal domain; RBD, receptor binding domain; S1/S2, S1/S2 protease cleavage site; S1, S1 Subunit; S2 ECD, spike S2 extracellular domain. The NAb titers were measured by the mSAIS assay and grouped in Figure 5.

To provide insight into how well the high-titer NAb samples could protect against SARS-CoV-2 variants, we analyzed the differential expression of anti-S antibodies of vaccinee serum using the SARS-CoV-2 proteome peptide microarray containing S proteins and tiled peptides representing the full-length S protein as previously described (Table S3) 19, 40. A z-score was used to identify antibodies that were differentially expressed between the high- and low-NAb titer groups. The calculated z-scores for antibodies that target the S protein's S1, S2 extracellular domain (ECD), and RBD were X, Y, and Z, respectively. In all three cases, the antibody levels were higher in the high-titer NAb group than in the low-titer NAb group. However, the differential expression of anti-RBD antibodies was the most pronounced across the two groups (Figure 6A).

Next, we performed epitope mapping of the vaccinees' serological anti-S antibodies. The results indicate that many of the antibodies that were produced targeted linear epitopes on the S protein (Figure 6B). Notably, the antibodies to the S-82 peptide (KPSKRSFIEDLLFNK), a fusion peptide previously identified as a target for COVID-19 diagnostics and neutralization 19, 41, was generated in 47% (8/17) and 85% (11/13) of low-titer and high-titer NAb group vaccinees, respectively.

In order to know the differential diversity of epitopes recognized by NAbs in high-titer and low-titer NAb vaccinee groups, we defined immunogenic peptides as having a z-score >1.96, and calculated the total number of nonredundant immunogenic peptides. The results showed that the total number of nonredundant immunogenic peptides within the S1, S2ECD, and RBD domains was consistently higher in the high-titer NAb group than the low-titer NAb group (Figure 6C).

**Figure 7:**
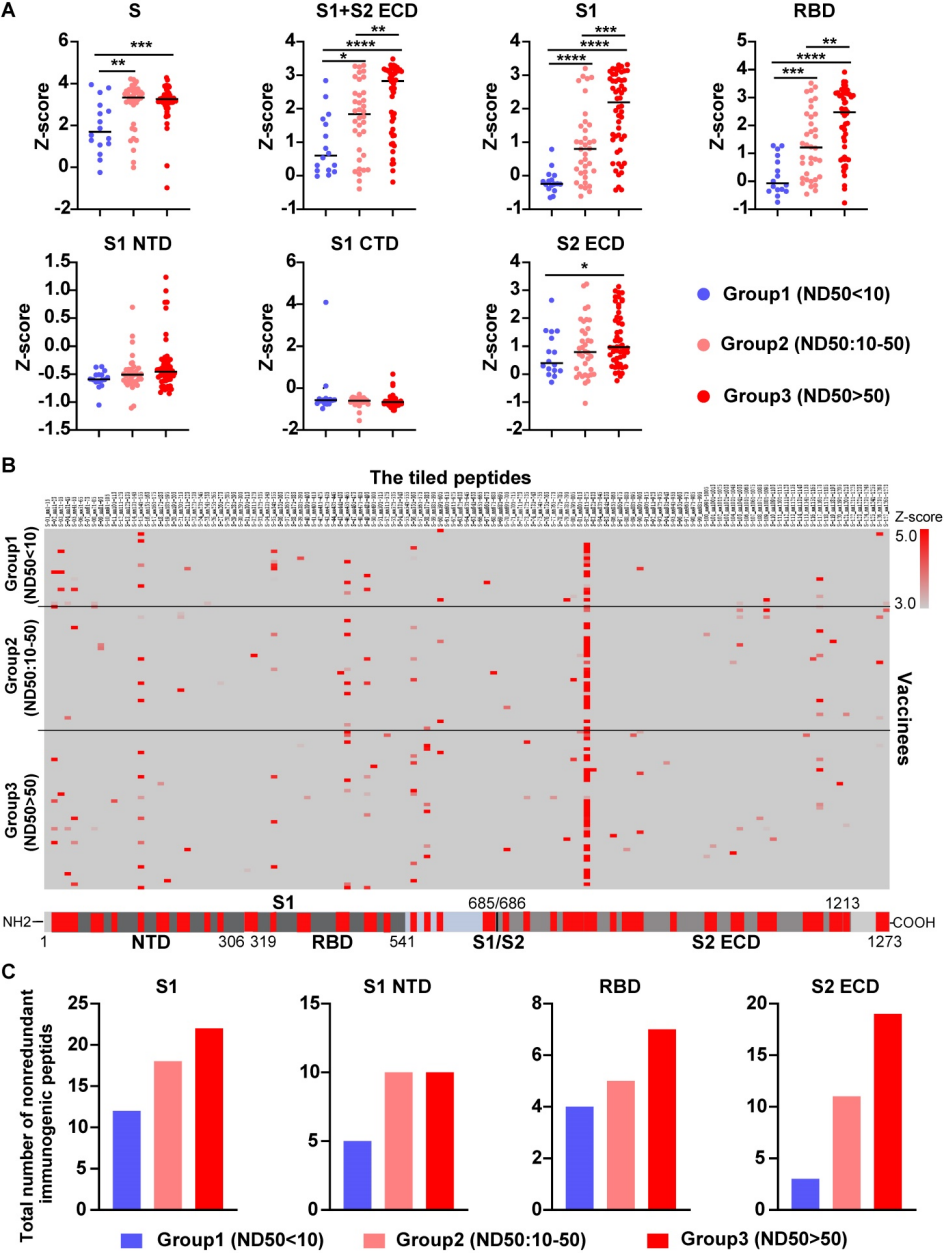
** Validation of anti-S antibody diversity in 104 vaccinees.** (A) A comparison of levels of antibodies that target the S proteins and its domains measured by the SARS-CoV-2 proteome microarray between group 1 (n = 16), group 2 (n = 36), and group 3 (n = 52) with different NAb titers. The data plotted were the mean with the error bars representing the standard deviation (SD). (B) Landscape of vaccine-induced IgG antibody epitopes on the S protein. (C) The total number of nonredundant immunogenic peptides on the S protein in the three groups. A p-value (p) ≤ 0.05 by Mann-Whitney test was significant. *, P < 0.05, **; P < 0.01; ***, P < 0.001; ****, P < 0.0001. NTD, N-terminal domain; RBD, receptor binding domain; CTD, C-terminal domain; S1/S2, S1/S2 protease cleavage site; S1, S1 Subunit; S2 ECD, spike S2 extracellular domain. The NAb titers (ND50) was measured using pseudovirus neutralization assay.

To further validate our findings, we measured the level of serological anti-S antibodies from 104 vaccinees who received the COVID-19 inactivated vaccine using SARS-CoV-2 proteome peptide microarray (Table S5). Prior to data analysis, all vaccinees were separated into three groups according to their NAb titers, which were measured by the SARS-CoV-2 pseudovirus neutralization assay (ND50<10, 10-50, >50). Notably, the levels of antibodies that target the S full protein, S1+S2 ECD, S1, S2 extracellular domain (ECD), and RBD were consistently higher in the high NAb titer group than the low-titer NAb group (Figure 6A and 7A). Via epitope mapping, we determined that the anti-S antibodies in the high-titer NAb group bind more peptides representing the S1, S1 N-terminal domain, RBD, and S2 ECD of the S protein than that of the low-titer NAb group (Figure 7B-C). These data indicate that high titer NAbs contain anti-S antibodies with more binding diversity. It is worth noting that the peptide microarray is unable to detect antibody binding to conformational epitopes; thus, all epitopes identified in this study are liner epitopes.

## Discussion

The SARS-CoV-2 virus mutated over time, resulting in circulating viral strains with altered transmission efficiencies, mortality rates, and S variants that can escape from the neutralizing effect of antibodies 7, 9, 21, 32. Therefore, significant effort has been devoted to improve the diagnosis, prevention, and treatment of COVID-19 patients infected with SARS-CoV-2 variants 42.

In this work, we developed an mSAIS assay that enables the detection of NAbs to numerous S variants simultaneously and rapidly. It has numerous advantages compared to the enzyme-linked immunosorbent assay (ELISA) and pseudovirus neutralization assays 28, 30, 43. First, our flexible platform can be easily adapted to include new S variants as they emerge. Second, only a laser scanner capable of detecting Cy5 is required to perform the mSAIS assay. Third, the multiplexed nature of the platform significantly reduces the sample volume, reagents, time, and cost to obtain data compared to single-plexed assays 44. For example, only 20 µL of serum was needed to screen for NAbs against 72 S variants in this study. In comparison, the volume requirements for ELISA or the pseudovirus neutralization assay would be 1,440 (72×) or 3,240 (162×) µL, respectively. Lastly, the mSAIS assay enables high throughput inhibitor screening to identify neutralizing antibodies to S variants without the need for a BSL3 laboratory.

The S variants tested here contain mutations that were selected from the COVID-19 virus mutation tracker database 21 and literature 22, 23, including known mutations like N501Y, L452R, K417N, N439K, S477N and S494P, and less publicized mutations (Figure S2). Comprehensive analyses of the effect of mutations on the neutralizing activity of antibodies will help understand the disease response at the individual and community levels following infection or COVID-19 vaccination. We recognize that the SARS-CoV-2 virus is continually evolving in a manner that cannot be accurately predicted.

Using the mSAIS assay, we mapped the resistance and sensitive mutations to three purified mouse and rabbit antibodies. We then evaluated their neutralizing activity to different S variants. Although these antibodies are capable of binding to RBD proteins (Figure S6), antibody #73 did not show neutralizing activity to all variants (Figure 3A). In comparison, antibodies #21 and #53 did not have any neutralizing activity to 8 and 5 variants, respectively, with 4 overlapped mutations (F456E, N487R, N501Y, Y505C). While N501Y is a known loss-of function mutation 45, 456E, N487R, and Y505C are new resistant mutations that should be assessed further. Such comprehensive mapping of antibodies to different S variants would be invaluable toward understanding mutation sensitivity to antibody neutralization and developing antibody cocktails for COVID-19 therapy 22, 30, 32, 46.

Notably, the N501Y mutation is present in several SARS-CoV-2 lineages and its role in COVID-19 is inconclusive. In Cheng's study, 12 monoclonal antibodies showed varying neutralizing levels against the N501Y mutant compared with wild-type SARS-CoV-2 47. Ding et al. identified a monoclonal antibody (03-1F9) with decreased neutralization activity to the N501Y mutant 48. It is possible that N501Y may induce neutralization resistance in the serum from convalescent COVID-19 patients 49 or the N501Y mutant may alter the availability of epitopes that are targeted by the antibodies. Moreover, resistance could be enhanced with the combination of E484K/K417N mutations 50-52. The results are consistent to our data using monoclonal and polyclonal antibodies and the serum from convalescent patients and vaccinees (Figure 4 and 5), suggesting that N501Y/E484K/K417N mutations improve the inhibition of S-ACE2 interactions.

Using serum from convalescent COVID-19 patients and vaccinees with the mSAIS assay, we identified 4 and 2 new resistant mutations, respectively, that escape NAb recognition (Figures 4 and 5). We also show that there are fewer escape mutations in high NAb titers than low titers, and this was observed in both convalescent COVID-19 patients and vaccinees (Figures 4C and 5C). Using a large cohort of 104 vaccinees with multiple assays (i.e., mSAIS assay, peptide array, pseudovirus neutralization assay), we further demonstrate that high titer NAbs contain anti-S antibodies that target more diverse binding epitopes, thus leading to more neutralizing capacity across a breadth of S variants (Figure 6 and 7). The production of heterogenous antibodies could be due to the somatic mutations that occur during antibody maturation 53. Finally, our data suggest that an effective COVID-19 vaccination strategy to defend against the mutating SARS-CoV-2 is to elicit the immune response to produce high-titer NAbs that contain a large diversity of anti-S antibodies 39, 54. However, the association between NAb titer and its ability to protect against SARS-CoV-2 infection in humans is unclear 55.

There were several limitations in this study. First, additional host factors [i.e., transmembrane protease, serine 2 (TMPRSS2), cathepsin L] other than ACE2 help facilitate viral entry and could be the targets for neutralization 56. Second, the mSAIS assay is *in vitro*, which might not accurately reflect results *in vivo*. Third, the conformational and glycosylation epitopes cannot be detected using the peptide microarray that employs chemically-synthesized peptides 19. Finally, the number of serum samples from convalescent COVID-19 patients and vaccinees were limited. The results obtained in this study should be validated in a large different cohort in the future.

## Conclusion

Altogether, we developed a high-throughput microarray-based mSAIS assay, which enables the multiplexed and rapid screening of NAbs to SARS-CoV-2 S variants. Using the mSAIS assay, we confirmed the neutralization capabilities of NAbs to known mutations, identified a number of new mutations that are resistant to the NAbs, and showed that there were fewer escape mutations with high titers NAb than low titers NAb in both convalescent COVID-19 patients and vaccinees. The data demonstrate the great potential of our proteomics platform in mapping the ability of NAbs to block the interaction between SARS-CoV-2 S variants and ACE2 interactions. Although we tested purified antibodies and serum in this study, the mSAIS assay could be used with other sample types as well. Data gleaned from the mSAIS will help develop more effective vaccines and therapeutic antibodies to fight against COVID-19.

## Materials and methods

### Collection of patient samples

Clinical specimens were obtained from the Department of Clinical Laboratory in Beijing Ditan Hospital, Capital Medical University (Beijing, China). Whole blood was collected in a vacutainer tube, and centrifuged at 4,000×g at room temperature (RT) for 10 min to separate serum. Serum was then transferred to a clean tube and stored at -80 °C until use. For safety purposes, serum collected from convalescent COVID-19 patients was inactivated at 56 °C for 30 min prior to any further sample processing. Notably, serum from COVID-19 vaccinated individuals was obtained from healthy hospital staff 4 weeks or 24 weeks after receiving the second vaccine dose, and convalescent samples were collected from COVID-19 patients 2 to 4 weeks post discharge. Our research was approved by the Ethics Committee of Beijing Ditan Hospital (No. 2021-010-01), and exemption of informed consent was obtained prior to sera collection.

### Expression and purification of recombinant proteins for use on the mSAIS assay

The DNA sequence encoding the SARS-CoV-2 Spike protein (S1+S2 ECD) (YP_009724390.1) (Val 16-Pro1213), S1 Subunit (YP_009724390.1) (Val16-Arg685), RBD (YP_009724390.1) (Arg319-Phe541) or variants were expressed with a polyhistidine tag at the C-terminus. The generation of variants using site-directed mutagenesis was performed as previously described 22. In addition, two negative controls [SARS-CoV-2 Nucleocapsid (N), MERS-CoV-2 Spike RBD], one positive control (SARS-CoV-2 wild-type RBD), and the extracellular domain of ACE2 were expressed with a polyhistidine tag at the C-terminus. The proteins were expressed in HEK293 cells, followed by further purification on a Ni-NTA spin column (Thermo Fisher). Protein purity was confirmed using an SDS-PAGE gel stained with Coomassie blue.

### Preparation of the SARS-CoV-2 spike variant protein microarray

All purified proteins were diluted to 100 µg/mL and printed onto a three-dimensional (3D)-modified slide surface (Capital Biochip Corp, Beijing, China) in parallel and in duplicate using an Arrayjet microarrayer (Roslin, UK) as previously described 19, 57, 58. The protein microarrays were stored at -20 °C until ready to use.

### Detection of NAbs using the mSAIS assay

Prior to antibody detection, the SARS-CoV-2 spike variant protein microarrays were assembled in an incubation tray and blocked with 1% (w/v) milk in 1x phosphate buffered saline, pH 7.2 (PBS), with 0.2% (v/v) Tween-20 (PBST) for 10 min at room temperature. After washing with PBST three times, the array was incubated with serum diluted 10- to 320-fold in PBST for 30 min or diluted antibodies for 1 h at room temperature. After washing again, the array was then incubated for 60 min with Cy5-labeled ACE2 (50 ng/mL). Finally, the array was washed with PBST and water, dissembled from the tray, and dried via centrifugation for 2 min at 2,000 rpm. The array was scanned with a GenePix 4300A microarray scanner (Molecular Devices, Sunnyvale, CA, USA) at a 10 μm resolution using a laser at 635 nm with 100% power/ PMT Gain 900. The median fluorescent signal intensity with background subtraction was extracted using GenePix Pro7 software (Molecular Devices, Sunnyvale, CA, USA). The neutralizing antibody titer of serum is expressed by the effective concentration (EC50). EC50 is the dilution of sera that inhibits 50% S-ACE2 binding in the mSAIS assay. EC50 was calculated using the GraphPad Prism software 8.3 based on the fluorescent signal intensity.

### SARS-CoV-2 pseudovirus neutralization assay

The SARS-CoV-2 pseudovirus was prepared by Beijing Gobond Testing Technology, Co. (Beijing, China) using the VSV-ΔG system in which the glycoprotein (G) gene was replaced with the firefly luciferase (Fluc) reporter gene. The S protein was overexpressed and displayed on the VSV pseudovirus. Following S-ACE2 interaction, the pseudovirus entered the host cell where the Fluc gene was transcribed and translated. The addition of luciferase substrate resulted in luminescence where the amount of luminescence is proportional to the level of pseudoviral entry. Additional information on how the SARS-CoV-2 pseudovirus neutralization assay was performed can be found in previous work 59. Briefly, human sera were diluted using 3-fold serial dilutions with a starting concentration of 1:10 (two per dilution). The diluted sera were added into 96-well plates in duplicate, followed by the SARS-CoV-2 pseudovirus at a concentration of 1300 median tissue culture infective dose (TCID50/mL). The sera and pseudovirus were incubated together at 37 °C for 1 h, and then monkey Vero cells were added at 2*10^4 cells/100 mL cells per well. The plates were incubated at 37 °C in a humidified atmosphere with 5% CO_2_ for 24 h and then the relative light unit (RLU) of chemiluminescence was read by a luminometer. The infection inhibition rates of each dilution are calculated using the RLU values. The inhibition rate enabled the calculation of the neutralizing antibody titer (ND50) by the Reed-Muench method.

### Live SARS-CoV-2 neutralization assay

A suspension of Vero cells suspension was prepared at a cell density of 2×10^5^/mL. Serum samples were diluted 4-fold in cell culture medium and inactivated at 56 °C for 30 min. The inactivated serum was serially diluted 2-fold with MEM medium, followed by the addition of cell culture medium containing 100 times the cell culture infective dose 50% (CCID50) of wild-type SARS-CoV-2 virus. The mixture was placed into a 96-well plate and incubated at 37 °C for 2 h in CO_2_ 5%, and then 2 × 10^4^ Vero cells were added and incubated again at 37 °C. The samples were microscopically examined for cytopathic effect (CPE) after 7 days. The highest dilution of serum that showed complete inhibition activity of SARS-CoV-2 was recorded as the neutralizing antibody titer (ID50). Assays were performed in duplicate with negative control serum.

The ID50 was calculated as: LogCCID50 = Xm + 1/2d - d ∑pi/100, where Xm is the log10 of maximum dilution of the virus, the d is the logarithm of the dilution fold, and ∑pi is the sum of the percentage of the cytopathic effect per dilution.

### Detection of SARS-CoV-2 serum antibodies using a SARS-CoV-2 proteome peptide microarray

The peptide microarray containing eight S proteins and tiled peptides representing the SARS-CoV-2 S protein was prepared as described in our previous work 19, 40. The array was assembled in an incubation tray and blocked with 5% (w/v) milk in phosphate-buffered saline (PBS) containing 0.05% (v/v) Tween-20 (PBST) for 30 min at room temperature before antibody detection. After aspirating the blocking solution, the 1:200 diluted serum was added to the array and incubated at room temperature for 20 min. After washing three times with PBST, the array was then incubated for 20 min with a Cy3 labelled donkey anti-human IgG(H+L) antibody (Jackson ImmunoResearch, USA) (2 μg/mL). Finally, the array was washed with PBST and deionized water, disassembled from the tray and dried with vacuum pump. The slide was scanned at 532 nm using a GenePix 4300A microarray scanner (Molecular Devices, Sunnyvale, CA, USA). The median fluorescent signal intensity of each spot with background subtraction was extracted using GenePix Pro7 software (Molecular Devices, Sunnyvale, CA, USA). The raw data for each peptide of the S protein were normalized to the z-score. The immunogenic peptides were defined as those with a z-score >1.96. Epitope identification via epitope mapping was performed as described in our previous work 60.

### Data analysis

The ability of the serological NAbs to inhibit the S-ACE2 interactions across the different S variants was visualized as a heatmap using the MultiExperiment Viewer software version 4.9. Statistical analyses were performed using the GraphPad Prism software 8.3 and Microsoft Excel with the unpaired t test and Mann-Whitney test. A p-value (p) ≤ 0.05 was considered to be significant. *, p < 0.05; **, p < 0.01; ***, p < 0.005; ****, p < 0.001. The 3D structure of the SARS-CoV-2 RBD and ACE2 complex (PDB ID code: 6M0J) was visualized using the VMD 1.9.3, and mutations were annotated in red.

## Supplementary Material

Supplementary figures and tables.
