# Supplementary material for: Inhibitor screening using microarray identifies the high capacity of neutralizing antibodies to Spike variants in SARS-CoV-2 infection and vaccination
Source: Theranostics. 2022 Feb 28;12(6):2519–34. doi: 10.7150/thno.67038 (PMC8965487; doi:10.7150/thno.67038)
Supplement: Supplementary file 1 — Supplementary figures and tables. [file thnov12p2519s1.pdf]

\- Supplementary Information-

Inhibitor screening using microarray identifies the high capacity of neutralizing antibodies to Spike variants in SARS-CoV-2 infection and vaccination

Xiaomei Zhang[1,8], Mei Zheng[2,3,5,8], Hongye Wang[1,8], Haijian Zhou[4,5,8], Te Liang[1,8], Jiahui Zhang[1], Jing Ren[1], Huoying Peng[1], Siping Li[1], Haodong Bian[1], Chundi Wei[1], Shangqi Yin[2,3,5], Chaonan He[2,3,5]. Ying Han[2,3,5], Minghui Li[4,5], Xuexin Hou[4,5], Jie Zhang[6], Liangzhi Xie[6], Jing Lv[7], Biao Kan[4,5,9], Yajie Wang[2,3,5,9], Xiaobo Yu[1,9]

Affiliations

[1] State Key Laboratory of Proteomics, Beijing Proteome Research Center, National Center for Protein Sciences-Beijing (PHOENIX Center), Beijing Institute of Lifeomics, Beijing, 102206, China.

[2] Department of Clinical Laboratory, Beijing Ditan Hospital, Capital Medical University, Beijing, 100102, China.

[3] Department of Research Ward, Beijing Ditan Hospital, Capital Medical University, Beijing, 100102, China.

[4] State Key Laboratory of Infectious Disease Prevention and Control, National Institute for Communicable Disease Control and Prevention, Chinese Center for Disease Control and Prevention, Beijing, 102206, China

[5] Joint Laboratory for Pathogen Identification of ICDC and Ditan Hospital, Beijing Ditan Hospital, Capital Medical University, Beijing, 100102, China

[6] Beijing Key Laboratory of Monoclonal Antibody Research and Development, Sino Biological, Inc., Beijing, 100176, China

[7] Gobond Testing Technology (Beijing) Co., Ltd., Beijing, 102629, China.

[8]These authors contributed equally to this work.

[9] Correspondence: xiaobo.yu@hotmail.com (X. Y.), wangyajie@ccmu.edu.cn (Y. W.), kanbiao@icdc.cn (B. K.)

15 Pages

5 Supplementary Tables

10 Supplementary Figures

Supplementary tables

Table S1	The worldwide cumulative prevalence of SARS-CoV-2 spike protein mutations in GISAID as of December 28, 2021
Table S2	Clinical information of convalescent COVID-19 patients
Table S3	Clinical information of patients in the study 4 weeks after receiving the second dose of COVID-19 inactivated vaccine
Table S4	Clinical information of patients in the study 24 weeks after receiving the second dose of COVID-19 inactivated vaccine
Table S5	Clinical information of validation cohort 4 weeks after receiving the second dose of COVID-19 inactivated vaccine

Supplementary figures

Figure S1	SDS-PAGE analysis of purified recombinant SARS-CoV-2 S variants.
Figure S2	Timeline of when the top 20 RBD mutations emerged.
Figure S3	Layout of SARS-CoV-2 spike variant protein microarray.
Figure S4	Reproducibility of protein microarray preparation.
Figure S5	Correlation of the mSAIS assay and live SARS-CoV-2 neutralization assay.
Figure S6	Detection of different NAbs binding to immobilized RBD protein using the mSAIS assay.
Figure S7	Structural analysis of spike mutations located at the

	RBD-ACE2 interaction interface that are resistant to antibody #21.
Figure S8	NAb titers to the wild type and variant S proteins in convalescent COVID-19 patients.
Figure S9	NAb titers to the wild type and variant S proteins in vaccinees.
Figure S10	Heat map of NAbs titers to S variants in vaccinees' sera after the second dose.

Supplementary tables

Table S1: The worldwide cumulative prevalence of SARS-CoV-2 spike protein mutations in GISAID as of December 28, 2021

Mutations	Mutation found		when found**	
	total	cumulative prevalence*	first	last
S1 (D614G)	6,239,957	99%	26-Jan-20	23-Dec-21
RBD (L452R)	3,749,640	59%	26-Jan-20	23-Dec-21
RBD (N501Y)	1,345,483	21%	7-Feb-20	23-Dec-21
S1 (A222V, D614G)	561,725	9%	2-Feb-20	23-Dec-21
S1 (L18F, D614G)	229,364	4%	28-Jan-20	20-Dec-21
RBD (S477N)	124,442	2%	1-Mar-20	17-Dec-21
S1 (T20N, D614G)	115,372	2%	28-Jan-20	23-Dec-21
RBD (K417N)	58,007	1%	16-Mar-20	14-Dec-21
RBD (N439K)	37,149	1%	15-Feb-20	23-Dec-21
RBD (K417N, E484K, N501Y)	33,998	1%	27-Mar-20	15-Dec-21
RBD (N440K)	20,878	< 0.5%	28-Jan-20	13-Dec-21
RBD (E484Q)	17,709	< 0.5%	27-Jan-20	14-Dec-21
RBD (S494P)	13,439	< 0.5%	3-Mar-20	7-Dec-21
RBD (G446S)	13,261	< 0.5%	5-Mar-20	14-Dec-21
RBD (G446V)	10,960	< 0.5%	19-Mar-20	19-Dec-21
RBD (A520S)	9,366	< 0.5%	18-Mar-20	18-Dec-21
RBD (S477I)	8,331	< 0.5%	10-Mar-20	20-Dec-21
RBD (A522V)	6,810	< 0.5%	26-Mar-20	12-Nov-21
RBD (P384L)	3,783	< 0.5%	25-Feb-20	16-Dec-21

RBD (V367F)	3,146	< 0.5%	13-Mar-20	18-Dec-21
RBD (A348S)	2,581	< 0.5%	21-Nov-21	21-Nov-21
RBD (L455F)	2,398	< 0.5%	15-Mar-20	20-Dec-21
RBD (Q414R)	2,026	< 0.5%	15-Mar-20	23-Dec-21
RBD (R408I)	1,984	< 0.5%	13-Mar-20	18-Dec-21
RBD (A475V)	1,967	< 0.5%	31-Mar-20	10-Dec-21
RBD (F490L)	1,701	< 0.5%	7-Apr-20	15-Dec-21
RBD (S477R)	1,567	< 0.5%	18-Mar-20	2-Dec-21
RBD (A352S)	1,518	< 0.5%	28-Mar-20	18-Dec-21
RBD (Y453F)	1,405	< 0.5%	1-Feb-20	14-Dec-21
RBD (Y508H)	1,195	< 0.5%	28-Jan-20	9-Dec-21
RBD (F338L)	1,126	< 0.5%	31-Mar-20	21-Nov-21
RBD (E471Q)	1,082	< 0.5%	6-Apr-20	6-Dec-21
RBD (T478I)	917	< 0.5%	3-Mar-20	18-Dec-21
RBD (N370S)	758	< 0.5%	30-Mar-20	8-Dec-21
RBD (P499R)	742	< 0.5%	18-Mar-20	14-Dec-21
RBD (V503F)	640	< 0.5%	28-Mar-20	20-Dec-21
RBD (A435S)	540	< 0.5%	2-Apr-20	15-Dec-21
RBD (S359N)	528	< 0.5%	25-Mar-20	18-Dec-21
RBD (F456L)	516	< 0.5%	8-Apr-20	3-Dec-21
RBD (V483I)	485	< 0.5%	29-Mar-20	29-Mar-20
RBD (V483A)	456	< 0.5%	15-Mar-20	14-Aug-21
RBD (N354D)	445	< 0.5%	29-Feb-20	17-Dec-21
RBD (E406Q)	372	< 0.5%	1-Mar-20	14-Dec-21
RBD (P521S)	338	< 0.5%	17-Mar-20	20-Dec-21
RBD (K378N)	310	< 0.5%	10-Apr-20	15-Dec-21
RBD (A520V)	304	< 0.5%	10-Mar-20	23-Dec-21
RBD (V445F)	266	< 0.5%	15-Apr-20	16-Dec-21
RBD (P337S)	242	< 0.5%	13-Mar-20	16-Dec-21
RBD (V341I)	229	< 0.5%	14-Jan-21	14-Dec-21
RBD (K458R)	181	< 0.5%	1-Apr-20	13-Dec-21
RBD (G485S)	176	< 0.5%	6-Apr-20	15-Dec-21
RBD (K378R)	88	< 0.5%	30-Mar-20	15-Dec-21
RBD (F377L)	87	< 0.5%	8-Oct-20	14-Dec-21
RBD (F342L)	74	< 0.5%	6-May-20	18-Dec-21
RBD (N481D)	67	< 0.5%	19-Feb-20	15-Dec-21
RBD (A372T)	61	< 0.5%	15-Dec-20	9-Dec-21
RBD (F486S)	46	< 0.5%	4-Apr-20	23-Dec-21
RBD (A372S)	44	< 0.5%	8-Jul-20	16-Dec-21
RBD (V395I)	26	< 0.5%	1-Apr-20	15-Dec-21
RBD (T385A)	22	< 0.5%	8-Jun-20	7-Dec-21
RBD (W436R)	7	< 0.5%	3-Mar-20	18-Dec-21
RBD (Q414E)	1	< 0.5%	18-Mar-20	19-Dec-21
RBD (T393P)	1	< 0.5%	29-Jul-20	15-Dec-21

S1 (N234Q)	0	not detected
S1 (HV69-70 deltion, N501Y, D614G)	0	not detected
RBD (Y505C)	0	not detected
RBD (F456E)	0	not detected
RBD (N487R)	0	not detected

Note: The prevalences were calculated by Outbreak.info Mutation Situation Reports (https://outbreak.info/situation-reports#voi) with 6,315,868 sequences from GISAID as of 28 December 2021.

* Apparent cumulative prevalence is the ratio of the sequences containing mutation to all sequences collected since the identification of mutation in that location.

** Dates are based on the sample collection date.

Table S2: Clinical information of the convalescent COVID-19 patients

Gender	n	Age, median	Weeks post discharge
Female	11	49 (18 ~ 64)	2 or 4
Male	14	36 (5 ~ 68)	2 or 4
Total	25	43 (5 ~ 68)	2 or 4

Table S3: Clinical information of people in the study 4 weeks after receiving the second dose of COVID-19 inactivated vaccine

Gender	n	Age, median	Weeks post the second vaccine dose
Female	6	36 (21 ~ 53)	4
Male	24	37 (23 ~ 57)	4
Total	30	37 (21 ~ 57)	4

Table S4: Clinical information of patients in the study 24 weeks after receiving the second dose of COVID-19 inactivated vaccine

Gender	n	Age, median	Weeks post the second vaccine dose
Female	18	38.5 (27 ~ 55)	24
Male	7	36 (31 ~ 46)	24
Total	25	38 (27 ~ 55)	24

Table S5： Clinical information of validation cohort 4 weeks after receiving the second dose of COVID-19 inactivated vaccine

Gender	n	Age, median	Weeks post the second vaccine dose
Female	11	46 (21 ~ 70)	4
Male	93	39 (23 ~ 70)	4
Total	104	39 (21 ~ 70)	4

Supplementary Figures

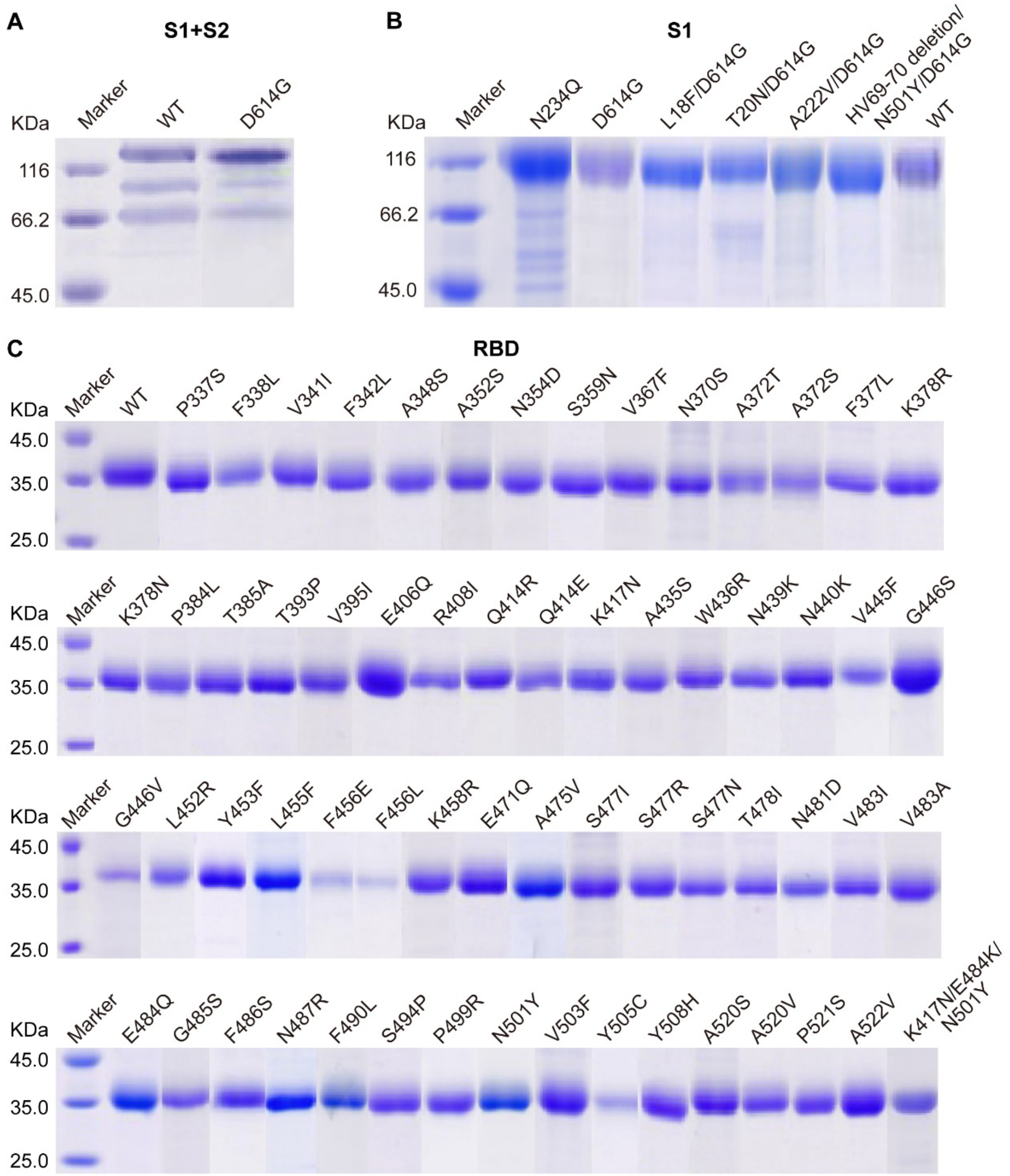

Figure S1. SDS-PAGE analysis of purified recombinant SARS-CoV-2 S variants.

(A-C) are SDS-PAGE analyses of S1+S2, S1 and RBD wild-type and variant proteins, respectively. WT: wild-type.

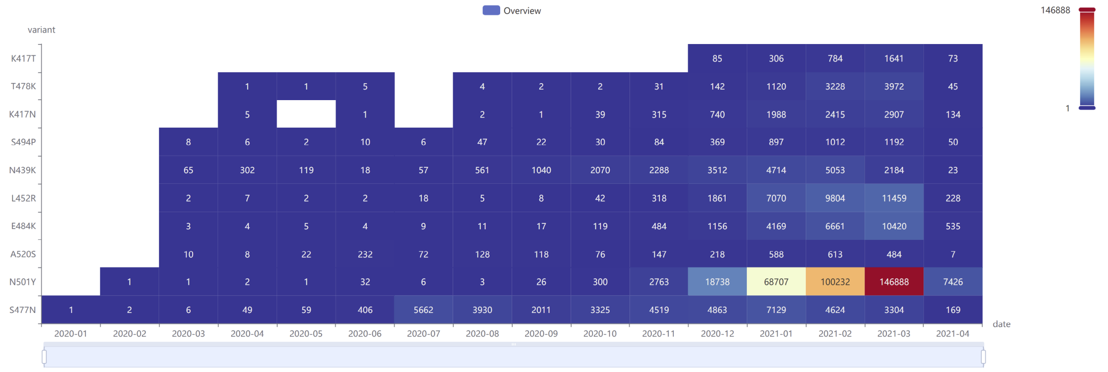

Figure S2. Timeline of when the top 20 RBD mutations emerged. The graph was obtained from the COVID-19 virus mutation tracker database (https://www.cbrc.kaust.edu.sa/covmt/index.php?p=top-rbd-variants-heatmap) [1].

	1	2	3	4	5	6	7	8	9	10	11	12
1	buffer	N	buffer	S1+S2	S1+S2(D614G)	S1	S1(N234Q)	S1(D614G)	S1(A222V/D614G)	S1(T20N/D614G)	S1(L18F/D614G)	S1(HV69-70 deltion/ N501Y/D614G)
2	buffer	N	buffer	S1+S2	S1+S2(D614G)	S1	S1(N234Q)	S1(D614G)	S1(A222V/D614G)	S1(T20N/D614G)	S1(L18F/D614G)	S1(HV69-70 deltion /N501Y/D614G)
3	RBD insect	RBD 293	RBD (V367F)	RBD(R408I)	RBD(V341I)	RBD(Y508H)	RBD(N439K)	RBD(V503F)	RBD(A522V)	RBD(S494P)	RBD(A372S)	RBD(N354D)
4	RBD insect	RBD 293	RBD (V367F)	RBD(R408I)	RBD(V341I)	RBD(Y508H)	RBD(N439K)	RBD(V503F)	RBD(A522V)	RBD(S494P)	RBD(A372S)	RBD(N354D)
5	RBD(A520S)	RBD(Q414E)	RBD(P384L)	RBD(A348S)	RBD(F338L)	RBD(F377L)	RBD(L452R)	RBD(P521S)	RBD(T478I)	RBD(V483I)	RBD(S359N)	RBD(K378R)
6	RBD(A520S)	RBD(Q414E)	RBD(P384L)	RBD(A348S)	RBD(F338L)	RBD(F377L)	RBD(L452R)	RBD(P521S)	RBD(T478I)	RBD(V483I)	RBD(S359N)	RBD(K378R)
7	RBD(A372T)	RBD(A520V)	RBD(A435S)	RBD(E406Q)	RBD(K378N)	RBD(N370S)	RBD(Q414R)	RBD(S477I)	RBD(S477N)	RBD(T385A)	RBD(T393P)	RBD(V395I)
8	RBD(A372T)	RBD(A520V)	RBD(A435S)	RBD(E406Q)	RBD(K378N)	RBD(N370S)	RBD(Q414R)	RBD(S477I)	RBD(S477N)	RBD(T385A)	RBD(T393P)	RBD(V395I)
9	RBD(V483A)	RBD(A475V)	RBD(G446V)	RBD(G485S)	RBD(N440K)	RBD(E471Q)	RBD(A352S)	RBD(K417N)	RBD(F342L)	RBD(P337S)	RBD(S477R)	RBD(L455F)
10	RBD(V483A)	RBD(A475V)	RBD(G446V)	RBD(G485S)	RBD(N440K)	RBD(E471Q)	RBD(A352S)	RBD(K417N)	RBD(F342L)	RBD(P337S)	RBD(S477R)	RBD(L455F)
11	RBD(K458R)	RBD(N481D)	RBD(F456L)	RBD(Y505C)	RBD(F456E)	RBD(F486S)	RBD(N487R)	RBD(G446S)	RBD(P499R)	RBD(V445F)	RBD(Y453F)	RBD(E484Q)
12	RBD(K458R)	RBD(N481D)	RBD(F456L)	RBD(Y505C)	RBD(F456E)	RBD(F486S)	RBD(N487R)	RBD(G446S)	RBD(P499R)	RBD(V445F)	RBD(Y453F)	RBD(E484Q)
13	RBD(N501Y)	RBD(F490L)	RBD(W436R)	RBD(K417N/ E484K/N501Y)	MERS-CoV RBD	SARS-CoV RBD	RBD-hFc	RBD-mFc	RBD-mFc (V367F)	RBD-rabbitFc	RBD	buffer
14	RBD(N501Y)	RBD(F490L)	RBD(W436R)	RBD(K417N/ E484K/N501Y)	MERS-CoV RBD	SARS-CoV RBD	RBD-hFc	RBD-mFc	RBD-mFc (V367F)	RBD-rabbitFc	RBD	buffer

Figure S3. Layout of SARS-CoV-2 spike variant protein microarray. The buffer and nucleocapsid (N) protein served as the negative controls. The RBD protein served as the positive control.

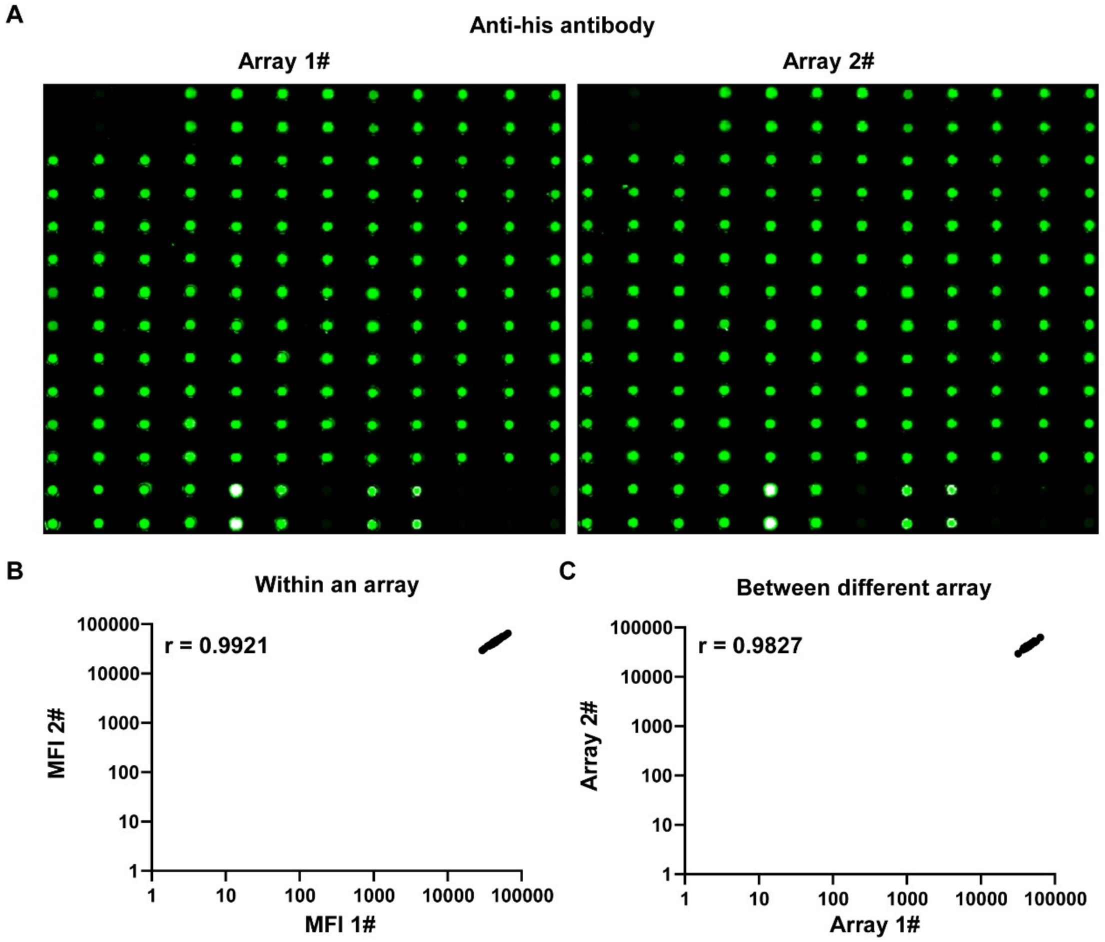

Figure S4. Reproducibility of protein microarray preparation. (A) Fluorescent staining of immobilized proteins on the glass-based microarray using an anti-his antibody; (B-C) are the intra- and inter-array reproducibility, respectively.

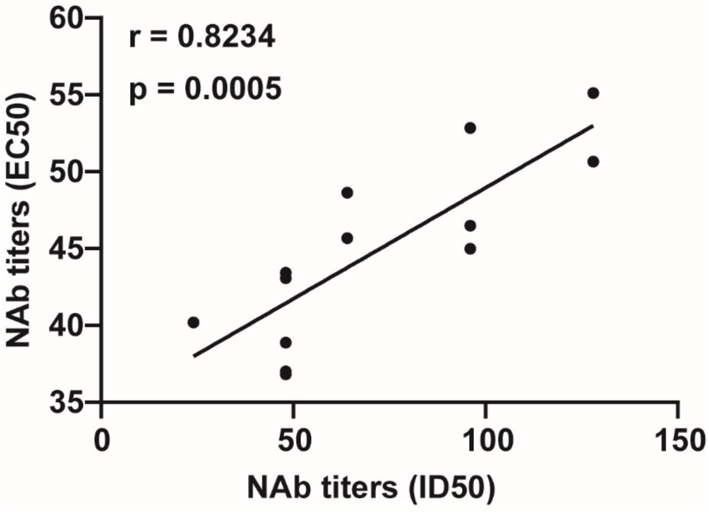

Figure S5. Correlation of the mSAIS assay and live SARS-CoV-2 neutralization assay. Pearson's correlation coefficient and linear regression analyses were performed by the GraphPad Prism software 8.3. Statistical significance was determined using the two-tailed t-test.

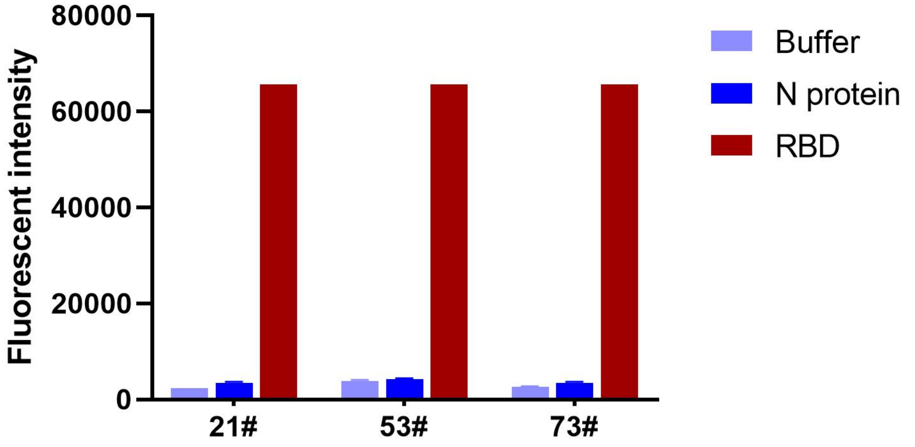

Figure S6. Detection of different anti-RBD antibodies binding to immobilized RBD protein using the SARS-CoV-2 proteome peptide microarray. The nucleocapsid (N) protein and buffer served as the negative controls.

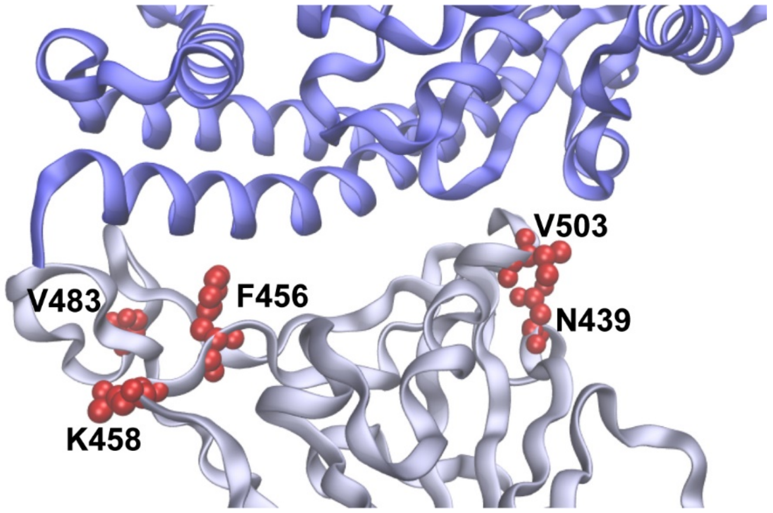

Figure S7. Structural analysis of spike mutations located at the RBD-ACE2 interaction interface that are resistant to antibody #21. The interaction structure of the SARS-CoV-2 RBD and human ACE2 were derived from the Protein Data Bank with PDB ID 6M0J. The S protein is colored in gray, the ACE2 protein is colored in blue, and RBD mutations are labeled in red.

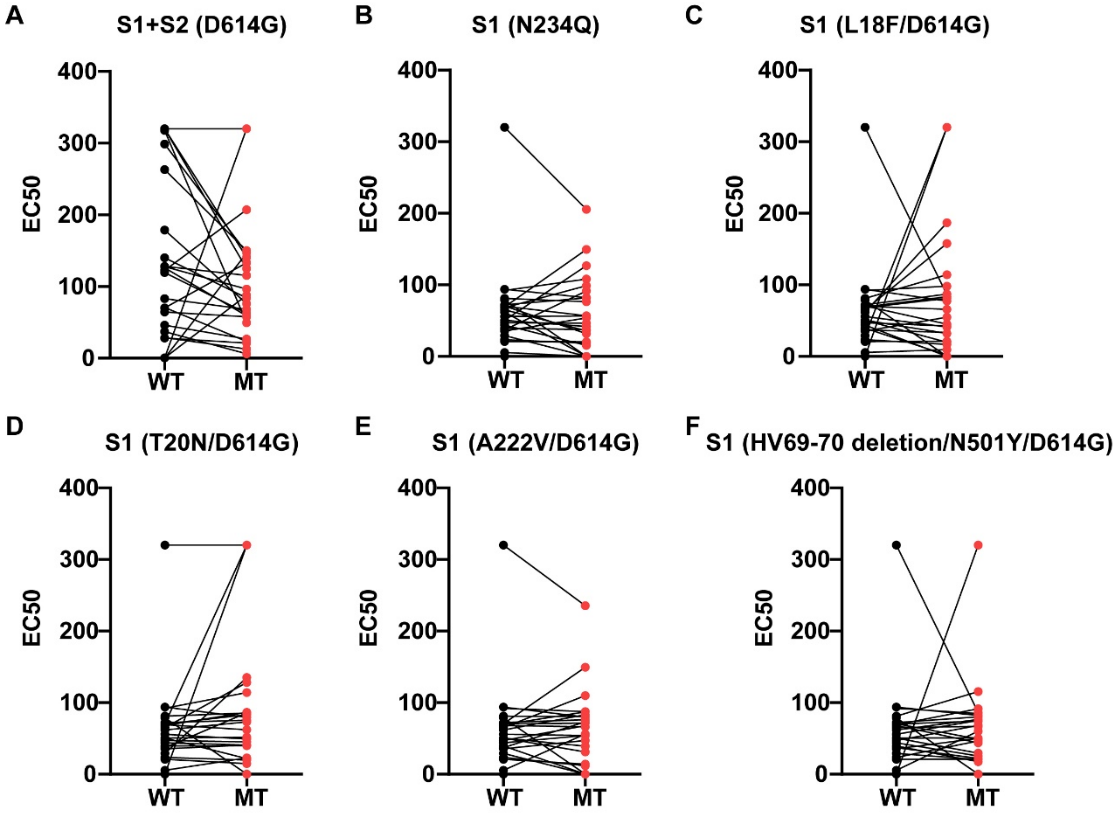

Figure S8. NAb titers to the wild type and variant S proteins in convalescent COVID-19 patients. (A-F) are the NAb titers of convalescent serum to different S variants with D614G, N234Q, L18F/D614G, T20N/D614G, A222V/D614G and hv69-70 deletion/N501Y/D614G, respectively. WT and MT represent the wild type and mutants, respectively.

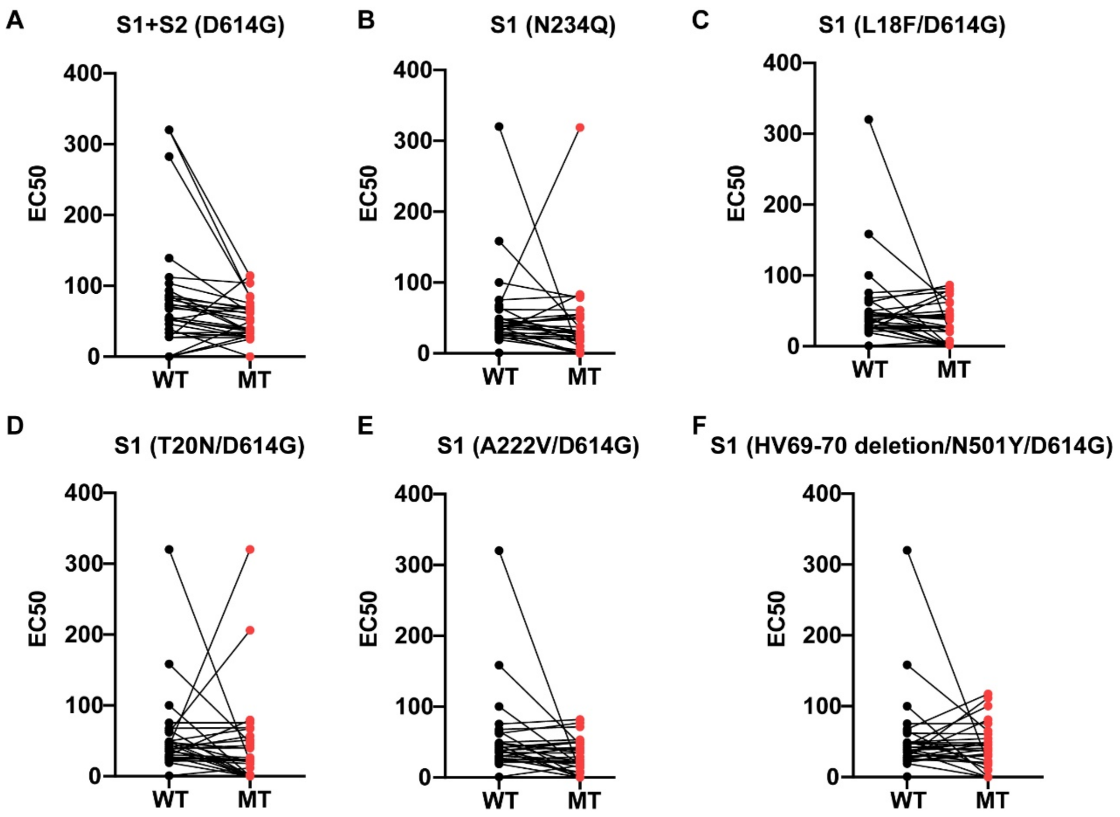

Figure S9. NAb titers to the wild type and variant S proteins in vaccinees. (A-F) are the NAb titers of vaccinees serum to different S variants with D614G, N234Q, L18F/D614G, T20N/D614G, A222V/D614G and hv69-70 deletion/N501Y/D614G, respectively. WT and MT represent the wild type and mutants, respectively.

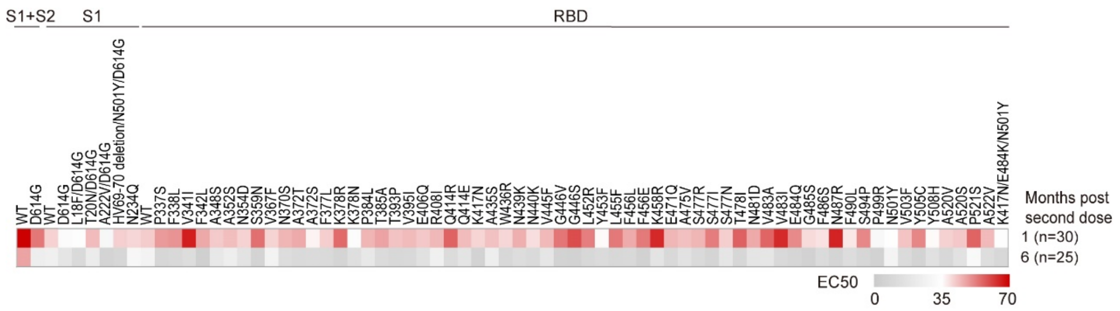

Figure S10. Heat map of NAbs titers to S variants in vaccinees' sera after the second dose. The NAb titers of 30 and 25 vaccinees one (1) and six (6) months, respectively, after receiving the second dose of the inactivated COVID-19 vaccine were measured using the mSAIS assay. The heat map was generated using the mean EC50. The rainbow color from gray to red correspond to the EC50 from low to high, respectively. WT = wild-type.

References:

1. Alam I, Radovanovic A, Incitti R, Kamau AA, Alarawi M, Azhar EI, et al. CovMT: an interactive SARS-CoV-2 mutation tracker, with a focus on critical variants. Lancet Infect Dis. 2021; 21: 602.
